# Health education services utilization and its determinants among migrants: a cross-sectional study in urban-rural fringe areas of Beijing, China

**DOI:** 10.1186/s12875-021-01368-1

**Published:** 2021-01-16

**Authors:** Shuang Shao, Huirong Zhang, Xiaolei Chen, Xiaojingyuan Xu, Yali Zhao, Meirong Wang, Juan Du

**Affiliations:** 1 School of General Practice and Continuing Education, Capital Medical University, Beijing, 100069 China; 2 Hospice Care Ward, Beijing geriatric hospital, Beijing, 100095 China

**Keywords:** Migrants, Health education utilization, Determinant factors, Anderson health service utilization behavioral model

## Abstract

**Background:**

Domestic migration poses a challenge for China as migrants have little access to preventive healthcare services and are vulnerable to certain risks and diseases. This research sought to unveil and explore the determinant factors associated with health education utilization as a key aspect in basic public health services for migrants in Beijing, China.

**Methods:**

A sample of 863 inter-provincial migrants, 18 years old and above, was selected by three-stage stratified cluster sampling method in urban-rural fringe areas of Beijing during 2016 to 2017. Face-to-face structured interviews were conducted in the questionnaire survey. The effects of the explanatory variables on health education utilization from predisposing, enabling, health behaviors and need variables were used to demonstrate by Anderson health service utilization model.

**Results:**

The study revealed that 61.6% migrants desired to receive health education, while only 53.8% of them received in the past year. There were differences in the utilization and needs of health education among the migrants in different ages and genders. Many migrants desired to gain access to various types of health education information from the internet. Chi-square independence test lists such major determinant factors in migrants whole health education as age, “*Hukou*” registration system, marital status, education level, long-term residence plan in Beijing, one or more children in Beijing, employment status, housing source, average daily working time, exercises, health knowledge, smoking, self-rated health. The binary logistic regression indicates that the migrants with younger age, high education level, one or more children in Beijing, exercises and good self-rated health were more likely to receive whole health education. The results also show that average daily working time of enabling variables and exercise of health behavior variables were the strong and consistent determinants of three types of health education utilization, including communicable, non-communicable and occupational diseases.

**Conclusion:**

Gaps exist between the needs and utilization in health education and more attention should be given to the migrants with heavy workload and low education level. Feasible policies and measures, such as multiple health information channels, should be vigorously implemented to ensure equitable and easy access to health education for migrants.

**Supplementary Information:**

The online version contains supplementary material available at 10.1186/s12875-021-01368-1.

## Background

Domestic migration poses major social, political and public health system challenges for cities in China. Migrants refer to individuals who move from the place where they born to other areas of the country without possessing the local “*Hukou*” (residence registration certificates), including inter-province or rural-to-urban migrants population. Past decades saw dramatically increasing migrants in China, reaching 245 million and constituting 18% of the total population in 2016 [1]. Although migrants are needed for socioeconomic development and urban construction in major cities, they often encounter several obstacles to accessing public services because of their distinguishing irregular characteristics (e.g. low wages, low education level, poor living condition, and insufficiently protected working environment) and economic and social marginalization (many public policies and social welfare programs were implemented based on a rigid “*Hukou*” system, serving as a domestic passport) [2, 3]. They suffer certain unnoticed health risks that can wear off their health awareness and make them vulnerable to health problems, such as communicable disease (CD), chronic non-communicable disease (NCD) and occupational disease, and also relatively easy to ignore their own health status [4, 5].

The real weakness of China’s public health system had been exposed after the outbreak of Severe Acute Respiratory Syndrome (SARS) in 2003 [6]. The Chinese government realized that health status and health awareness of public population migrants in particular, had a considerable impact on Chinese social stability and public health [7], and the fragmentary public health prevention and intervention systems should be re-established to control the spread of diseases and reduce the waste of medical resources [8]. To promote the gradual equalization of basic public health services, and to deepen Chinese healthcare reform, the program of National Basic Public Health Services was implemented in 2009 and provided the services to residents [9]. The basic public health services were free and voluntary public services were provided for permanent residents by primary health care institutions, including village clinics, township health centers, and community health service centers (stations), focusing on preventing and controlling diseases by public health intervention measures. At present, the basic public health services included fourteen basic items, including the establishment of health records, health education, vaccine inoculation, children and maternal health management, tuberculosis and hypertension management. Chinese government also raised the subsidy from 15 *Renminbi* (RMB) per capita in 2009, 25 RMB in 2011 to 45 RMB in 2016 for financing this project.

It cannot be denied that the rate of basic public health service utilization has increased rapidly among community permanent residents. The effectiveness of services also has remarkably improved (e.g., lives saved, suffering reduced and ill health improved) with more vigourous national support [10]. However, migrants are difficult to enter the basic public health service network due to their frequent mobility. An imbalance of basic public health services utilization does exist between the local and migrant population, for example, migrants, compared with local residents, have lower health awareness [11], higher rate of spread of CDs [12, 13]. Additionally, the migration trend has changed from “temporary residence” and “migrant alone” to “long-term residence” and “migration with family members” in the last decade [14]. With the change of migration model, diversified requirements of public health services should be met for migrants.

If the needs and utilization of basic public health services for them are not guaranteed, there will be a series of social problems as well as a potential threat to the local residents’ health. The Health Sector Reform formulated a set of strategies to “build up a strong basic public health service network” to promote the equalization of basic public health services. Since 2014, the National Health and Family Planning Commission (renamed National Health Commission of the People’s Republic of China from 2017) launched trials on the basic public health services equalization to improve migrants’ health in 40 cities across the country [15]. Put simply, providing accessible and good-quality basic public health services for migrants is an important issue.

Health education scheme, part of the Basic Public Health Service Program in China, targets at general population provided by community health service institutions (CHSIs), and has five forms, including “materials of health education”, “health education bulletin board”, “public health consultation”, “health knowledge lecture”, and “individualized health education”. Health education intervention and materials have proven to be an effective strategy for strengthening health knowledge, awareness and positive health behaviors. Furthermore, health education is essential for improving CDs and NCDs prevention, control, and treatment for general public and for the target group, particularly in the marginalized and migrant populations [16–18]. It is also likely to be instrumental in effectively addressing growing health care costs and in preventing or mitigating the negative effects of migration on health systems and society. While previous study found that compared with relatively high use of medical care, preventive care was used less frequently among migrants [19]. Study in Xi’an indicated that more than 50% migrants have not received occupational safety and health protection training [20]. A survey in Shanghai showed that in 2014, the rate of utilizing infectious disease health education was only about 30% among the migrants [21]. Migrants working in small- and medium-sized enterprises are at higher risk, due to the deficiency of occupational disease health education and supervision, compared with those in large-sized enterprises [22]. Moreover, current contents and traditional face-to-face education of health information ignore the actual utilization situation and fail to meet the needs of the general public with an increasing sense of health [23]. In order to implement successful policies to address social and health inequalities among the migrant populations, policy makers need to understand what barriers migrants face, and also need to identify and answer their health needs.

As the political, economic and cultural center of China, Beijing attracts tens of thousands of migrants from all over the country every year [24]. Systematic research on health education utilization behaviors and influence factors of migrants is far from sufficient. This research results are crucial to disease prevention and health promotion for the migrants in China. We aim to (1) evaluate differences between utilization behaviors and needs of health education; (2) put forth the potential major determinant factors of health education utilization behaviors in different sociodemographic, health behavior and health outcome setting for migrants in Beijing by using the simplified Anderson health service utilization behavioral model. The comparisons and inferences could help us figure out the obstacles in migrants health education, and take targeted intervention measures to improve health literacy, to control disease and to promote health status.

### Analytic framework

**Fig. 1:**
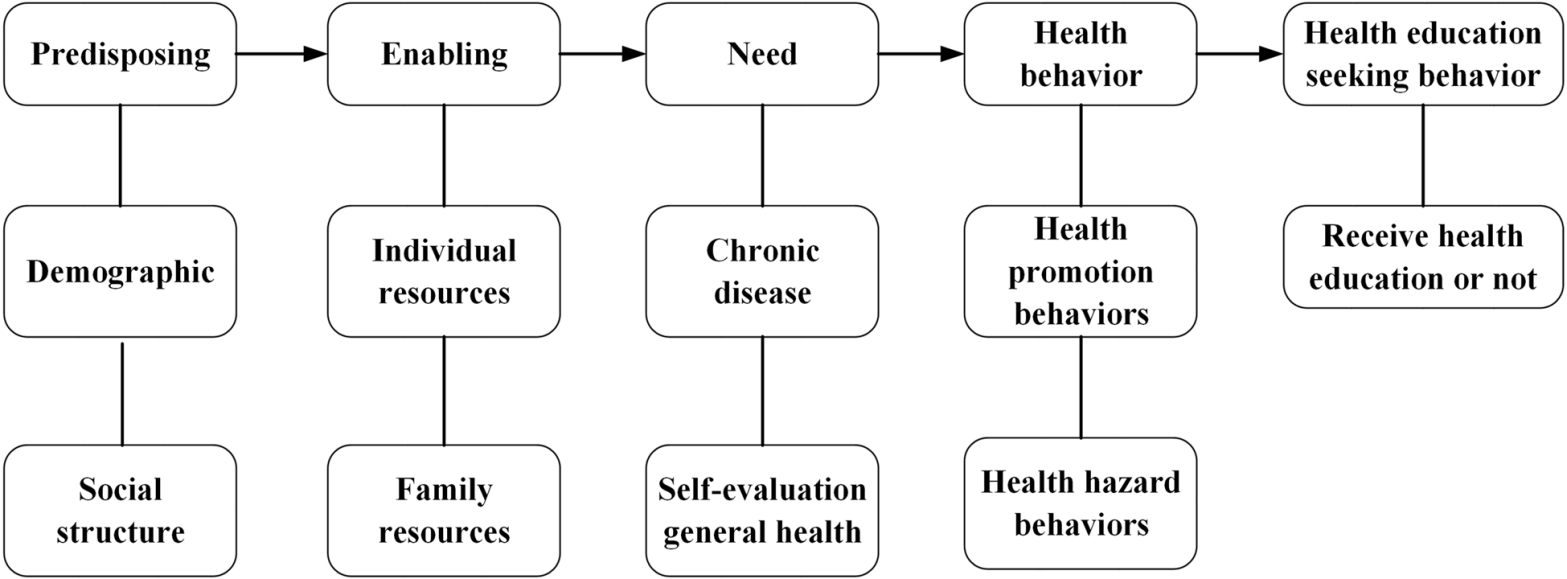
The simplified Anderson health education utilization behavioral model. Health education seeking behavior (seek health service or not) is determined by four dynamics: predisposing variances (demographic and social structure), enabling variances (individual and family resources), health behavior variances (health promotion behaviors and health hazard behaviors), and need variances (chronic disease and self-evaluation general health)

Andersen health service utilization behavioral model, a well-validated theoretical framework, can predict determinants of health services utilization, take into consideration both individual and societal determinants [25]. In the original model, health education utilization is determined by three dynamics: predisposing, enabling, and need variances (PEN). Social demographic characteristics including sex, age, race, etc., can be divided into predisposing factors, which increase one’s needs for health education services. For example, a person with strong belief in health education services for effective disease prevention is more likely to seek health education. Enabling factors are individual, family and community resources support, and can facilitate or impede the use of health education services. Need factors represent both actual and self-perceived needs for health education services. Based on the previous research [26] and the evolution of Andersen’s health services utilization behavioral model [27], health behavior variances were also considered as the determinants of accessing to health care. So, in our study, health behavior variances served as a key dynamics parameter and were integrated to evaluate the predictions of a new model. Also we used a feedback loop to illustrate the relationship between health education behaviors (seek health education, and not seek health education) and other aspects. (See Fig. 1).

## Methods

### Ethics statement

The study was undertaken as a part of Beijing Philosophy and Social Science Planning Project, a population-based cross-sectional survey on risk factors of health status for migrants. It was approved by the Ethical Committee of Capital Medical University, Beijing, China. Data were collected from a cross-sectional survey in urban-rural fringe areas of Beijing during 2016 to 2017. The parents or guardians are main decision-makers in public health services for children, thus those children under 18 were not included in this study. Written informed consent was obtained from each participant involved in this study. All participants’ information was anonymized and kept confidential.

### Data acquisition and study population

**Fig. 2:**
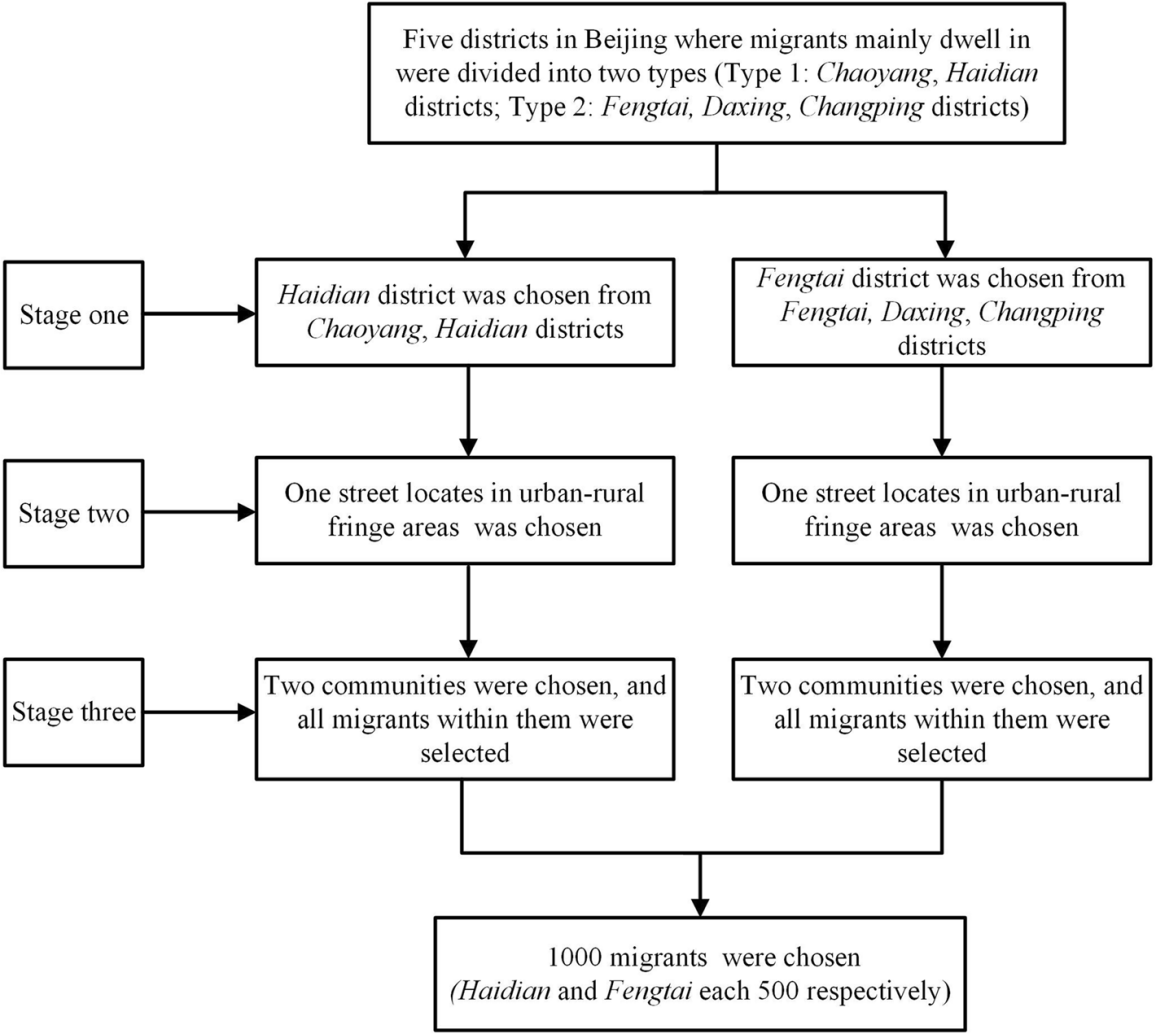
The sampling flow of migrants in urban-rural fringe areas in Beijing. A sample of 1000 migrants was selected by three-stage stratified cluster random sampling

A fieldwork survey of Public Health Service Utilization of Migrants Population in Beijing Urban-Rural Fringe Areas was performed from June 2016 to January 2017. All respondents were at least 18 years old, including inter-provincial migrants residing or working in the sampling regions (for no less than 6 months). Exclusion criteria consisted of the following: migrants who were not able to respond, those with mental health issues, and tourists in Beijing. The migrants dwell mainly in 5 (*Chaoyang*, *Haidian*, *Fengtai, Daxing*, *Changping* districts) out of 16 districts in Beijing. Five districts were divided into two types of region based on the number of migrants, including the region with more than 1 million migrants (*Chaoyang*, *Haidian* districts) and the region with 0.5 to 1 million migrants *(Fengtai, Daxing*, *Changping* districts). A sample of 1000 migrants was chosen from two of the five districts in Beijing by using three-stage stratified cluster random sampling, as follows: Stage One, one district was chosen from the first region (*Haidian* district), and the other district was chosen from the second region (*Fengtai* district). Stage Two, one street located in urban-rural fringe areas was chosen from each sampled district respectively according to the population size and social economic status. Stage Three, within each selected street, a list of residential communities (clusters) to allow for non-participation was compiled for migrants. Then two clusters were randomly selected from the list in each sampled street respectively. All migrants within the selected clusters were screened for eligibility and invited to participate in the study. The total number of 1000 migrants was recruited and investigated from four residential communities, with *Haidian* and *Fengtai* each 500 respectively. (See Fig. 2).

Data were collected via face-to-face cross-sectional surveys, and 921 migrants took part in the investigation. Finally, a total of 863 respondents were analyzed after excluding the data missing information on any of variables in the research. These samples can be considered representative of migrants population in Beijing as they presented similar distribution of age and gender status compared with those in the larger population, according to the report on China’s migrant population development in 2015 [28].

The structured questionnaire includes four parts of variances as follows: predisposing factors (sociodemographic characteristics), enabling factors (individual/family resources), need factors (general health status), health behavior factors (health promotion behaviors and health hazard behaviors) and health education seeking behavior (receiving health education or not).

In this survey, utilization and needs of health education were measured by responses of three questions:

1. Have you received any form of health education in the past year?

The health education scheme of Basic Public Health Service Program includes five main forms, including receipt of “materials of health education”, “health education bulletin board”, “public health consultation”, “health knowledge lecture”, and “individualized health education” by population from CHSIs.

-Binary: 1 for received any form of health education at least once, 0 for did not receive.

2. What was/were the type/types of health education have you received in the past year?

The types of health education mainly consisted of “occupational disease prevention and therapy”, “child healthcare”, “antenatal, prenatal and postpartum healthcare”, “CD prevention and therapy”, “NCD prevention and therapy”, “adolescent healthcare”, “menopause healthcare” and “aged healthcare”, etc. Respondents should answer the question according to their utilization of health education.

3. What is/are the type/types of health education that you want to receive in the future?

The types of health education were same as in the question two. Respondents should answer the question according to their needs.

Quality-assurance measures for this survey include questionnaire evaluation, training investigators, and fieldwork supervision to monitor the survey procedure. It was reviewed, edited, and validated by experts from health administration and CHSIs prior to implementation. A trial survey covering 50 persons was implemented during June 6 to 11, 2016 for better understanding the questionnaire and the fieldwork procedures. Double entry and validation were adopted for all data using EpiData software (Version 3.1, EpiData Association, Odense, Denmark). Discrepancies, compared and analyzed from the two databases, would be clarified by reviewing the original data source.

### Statistical analysis

A person was the unit of seeking health education at least once in the past year, rather than total number of times health education received. Chi-square independence test was used to analyze the differences in the categorical variables. Odds Ratios (ORs) and their 95% confidence intervals (CIs) were calculated using binary logistic regression analysis. In the first step, descriptive statistics and chi-square independence test were calculated, stratified by the categories of health education utilization (received and did not receive health education). In the second step, multivariate logistic regression analysis was performed to predict the potential major determinant factors in migrants health education utilization, and in possible confounding variables control. The full model consisted of all predisposing, enabling, health-related behaviors and the need factors were entered into the model. The full list of independent variables is summarized in the Table 1. All statistical analyses were performed using IBM Statistical Package for Social Science version 20.0 (SPSS Inc., Chicago IL, US) and all tests are two sided. The significance level for all analyses was set at *P* < 0.05.

**Table 1:** The list of variables for empirical analysis

**Predisposing**	**Demography**	**Age**	18–24 years (Reference group); 25–34; 35–44; 45–54; ≧55
		**Gender**	Male (Reference group); Female
	**Social structure**	**Marital status**	Unmarried (Reference group); married; divorced/widowed
		**Education level**	Primary school or below (Reference group); Junior high school; Senior high school; University or college and above
		**Ethnicity**	Han ethnic (Reference group); Minorities
		**“*Hukou*” registration system**	Non-agricultural (Reference group); Agricultural
**Enabling**	**Individual resources**	**Time in Beijing**	< 1 year (Reference group); 1-; 5-; ≧10
		**Plan to reside for a long time in Beijing**	No (Reference group); Yes
		**Employment status**	Formal work (Reference group); Informal work
		**Income monthly**	< 3000 RMB (Reference group); 3000–4999; 5000–9999; ≧10,000
		**Insurance status**	Uninsured (Reference group); Insured
		**Average daily working time**	< 8 h (Reference group); 8; > 8
	**Family resources**	**Have at least one child in Beijing**	No (Reference group); Yes
		**Housing source**	Own house (Reference group); Rent
**Health behavior**	**Health promotion behaviors**	**Do exercises**	No (Reference group); Yes
		**Acquire health knowledge**	No (Reference group); Yes
	**Health hazard behaviors**	**Smoking**	No (Reference group); Yes
		**Drinking**	No (Reference group); Yes
**Need**	**Having chronic disease**		No (Reference group); Yes
	**Self-evaluation general health status**		Good (Reference group); General; Poor

## Results

### Utilization of health education in the past year

In this study, 61.6% migrants desired to receive health education, while only 53.8% of them received it. Sample characteristics were presented in Table 2. A total of 863 inter-provincial migrants (males 383; females 480) in Beijing above 18 years old were investigated. The age of migrants varies from 18 to 76 years old, and average age was 36.6 ± 11.2 years old.

**Table 2:** Information on health education received by migrants in different characteristics

Variances	Total	Migrants (***N*** = 863)			
		Received health education n (%)	Did not receive health education n (%)	χ2	***P***
***Predisposing variables***					
**Gender**				0.022	0.882
Male	383(44.4)	207(44.6)	176(44.1)		
Female	480(55.6)	257(55.4)	223(55.9)		
**Age**				82.167	< 0.001
18–24	110(12.7)	84(18.1)	26(6.5)		
25–34	316(36.6)	207(44.6)	109(27.3)		
35–44	211(24.4)	97(20.9)	114(28.6)		
45–54	168(19.5)	56(12.1)	112(28.1)		
≧55	58(6.7)	20(4.3)	38(9.5)		
**Ethnicity**				1.667	0.197
Han	834(96.6)	445(95.9)	389(97.5)		
Minorities	29(3.4)	19(4.1)	10(2.5)		
**“*Hukou*” registration system**				20.025	< 0.001
Non-agricultural	248(28.7)	163(35.1)	85(21.3)		
Agricultural	615(71.3)	301(64.9)	314(78.7)		
**Marital status**				33.125	< 0.001
Unmarried	167(19.4)	123(26.5)	44(11.0)		
Married	680(78.8)	334(72.0)	346(86.7)		
Divorced/Widowed	16(1.9)	7(1.5)	9(2.3)		
**Education level**				97.200	< 0.001
Primary school or below	82(9.5)	18(3.9)	64(50.0)		
Junior high school	305(35.3)	124(26.7)	181(45.4)		
Senior High school	198(22.9)	121(26.1)	77(19.3)		
University or college and above	278(32.2)	201(43.3)	77(19.3)		
***Enabling variables***					
**Income monthly**				4.951	0.175
< 3000 RMB	191(22.1)	93(20.0)	98(24.6)		
3000–4999	321(37.2)	174(37.5)	147(36.8)		
5000–9999	284(32.9)	154(33.2)	130(32.6)		
≧10,000	67(7.8)	43(9.3)	24(6.0)		
**Time residing in Beijing**				6.555	0.088
< 1 year	82(9.5)	50(10.8)	32(8.0)		
1-	180(20.9)	83(17.9)	97(24.3)		
5-	288(33.4)	156(33.6)	132(33.1)		
≧10	313(36.3)	175(37.7)	138(34.6)		
**Plan to reside for a long time in Beijing**				49.602	< 0.001
No	331(38.4)	128(27.6)	203(50.9)		
Yes	532(61.6)	336(72.4)	196(49.1)		
**Have at least one child in Beijing**				28.977	< 0.001
No	639(74.0)	309(66.6)	330(82.7)		
Yes	224(26.0)	155(33.4)	69(17.3)		
**Employment status**				29.977	< 0.001
Formal work	349(40.4)	227(48.9)	122(30.6)		
Informal work	514(59.6)	237(51.1)	277(69.4)		
**Housing source**				7.529	0.006
Own house	104(12.1)	69(14.9)	35(8.8)		
Rent	759(87.9)	395(85.1)	364(91.2)		
**Insurance**				0.407	0.523
Uninsured	37(4.3)	18(3.9)	19(4.8)		
Insured	826(95.7)	446(96.1)	380(95.2)		
**Average daily working time**				43.817	< 0.001
< 8 h	31(3.6)	16(3.4)	15(3.8)		
8	402(46.6)	264(56.9)	138(34.6)		
> 8	430(49.8)	184(39.7)	246(61.7)		
***Health behavior***					
**Health promotion behaviors**					
**Do exercises**				29.872	< 0.001
No	494(57.2)	226(48.7)	268(67.2)		
Yes	369(42.8)	238(51.3)	131(32.8)		
**Acquire health knowledge**				24.476	< 0.001
No	417(48.3)	188(40.5)	229(57.4)		
Yes	446(51.7)	276(59.5)	170(42.6)		
**Health hazard behaviors**					
**Smoking**				13.121	< 0.001
No	710(82.3)	402(86.6)	308(77.2)		
Yes	153(17.7)	62(13.4)	91(22.8)		
**Drinking**				1.487	0.223
No	767(88.9)	418(90.1)	349(87.5)		
Yes	96(11.1)	46(9.9)	50(12.5)		
***Need variables***					
**Having chronic disease**				0.064	0.801
No	691(80.1)	373(80.4)	318(79.7)		
Yes	172(19.9)	91(19.6)	81(20.3)		
**Self-evaluation general health status**				24.467	< 0.001
Good	225(26.1)	128(27.6)	97(24.3)		
Moderate	379(43.9)	203(43.8)	176(44.1)		
Poor	259(30.0)	133(28.7)	126(31.6)		

Descriptive statistics and chi-square independence test were used to describe the information and to analyze the influence factors of health education utilization by sociodemographic factors. Chi-square independence test showed that age, **“***Hukou*” registration system, marital status, education level, plan to reside for a long time in Beijing, have at least one child in Beijing, employment status, housing source, average daily working time, do exercises, health knowledge, smoking, self-evaluation general health status are the major determinants affecting migrants to receive health education. (See Table 2).

Gender, “*Hukou*” registration system, education level, plan to reside for a long time in Beijing, have at least one child in Beijing, employment status, housing source, average daily working time, do exercises, acquire health knowledge are the major determinants affecting migrants to receive CD health education. “*Hukou*” registration system, housing source, average daily working time, do exercises are the major determinants affecting migrants to receive NCD health education. Age, **“***Hukou*” registration system, marital status, education level, plan to reside for a long time in Beijing, have at least one child in Beijing, employment status, average daily working time, do exercises, acquire health knowledge, smoking, are the major determinants affecting migrants to receive occupational disease health education. (See Table 3) Table 4 shows that the top five types of health education received by male migrants were “occupational disease prevention and therapy” (19.1%), “child healthcare” (14.9%), “antenatal, prenatal and postpartum healthcare” (10.2%), “CD prevention and therapy” (9.9%), and “NCD prevention and therapy” (9.1%). However, the top five types of health education that male migrants wanted to receive were “NCD prevention and therapy” (58.2%), “CD prevention and therapy” (46.7%), “child healthcare” (44.9%), “aged healthcare”(42.0%) and “occupational disease prevention and therapy” (36.8%). The top five types of health education received by female migrants were “antenatal, prenatal and postpartum healthcare” (18.8%), “child healthcare” (16.5%), “CD prevention and therapy” (14.4%), and “NCD prevention and therapy” (12.7%), “occupational disease prevention and therapy” (11.9%). However, the top five types of health education that female migrants wanted to receive were “NCD prevention and therapy” (63.5%), “aged healthcare”(51.7%), “CD prevention and therapy” (50.6%), “child healthcare” (48.3%) and “adolescent healthcare” (40.2%).

**Table 3:** Information on the three types of health education received by migrants in different characteristics

Variances	Migrants (N = 863)												
	Received CD health education n (%)	Did not receive CD health education n (%)	χ2	***P***	Received NCD health education n (%)	Did not receive NCD health education n (%)	χ2	***P***	Received occupational disease health education n (%)	Did not receive occupational disease health education n (%)		χ2	***P***
***Predisposing variables***													
**Gender**			3.890	0.049			2.746	0.097				0.022	0.882
Male	38(35.5)	345(45.6)			35(36.5)	348(45.4)			73(56.2)	310(42.3)			
Female	69(64.5)	411(54.4)			61(63.5)	419(54.6)			57(43.8)	423(57.7)			
**Age**			5.964	0.202			3.967	0.411				82.167	< 0.001
18–24	17(15.9)	93(12.3)			7(7.3)	103(13.4)			34(26.2)	76(10.4)			
25–34	41(38.3)	275(36.4)			35(36.5)	281(36.6)			53(40.8)	263(35.9)			
35–44	30(28.0)	181(23.9)			26(27.1)	185(24.1)			27(20.8)	184(25.1)			
45–54	12(11.2)	156(20.6)			19(19.8)	149(19.4)			13(10.0)	155(21.1)			
≧55	7(6.5)	51(6.7)			9(9.4)	49(6.4)			3(2.3)	55(7.5)			
**Ethnicity**			0.836	0.565			2.777	0.096				1.667	0.256
Han	105(98.1)	729(96.4)			90(93.8)	744(97.0)			125(96.2)	709(96.7)			
Minorities	2(1.9)	27(3.6)			6(6.3)	23(3.0)			5(3.8)	24(3.3)			
**“*Hukou*” registration system**			9.148	0.002			8.818	0.003				20.025	< 0.001
Non-agricultural	44(41.1)	204(27.0)			56(58.3)	208(27.1)			51(39.2)	197(26.9)			
Agricultural	63(58.9)	552(73.0)			40(41.7)	559(72.9)			79(60.8)	536(73.1)			
**Marital status**			2.749	0.253			3.520	0.172				33.125	< 0.001
Unmarried	18(16.8)	149(19.7)			16(16.7)	151(19.7)			56(43.1)	111(15.1)			
Married	85(79.4)	595(78.7)			76(79.2)	604(78.7)			72(55.4)	608(82.9)			
Divorced/Widowed	4(3.7)	12(1.6)			4(4.2)	12(1.6)			2(1.5)	14(1.9)			
**Education level**			8.404	0.038			1.870	0.600				97.200	< 0.001
Primary school or below	5(4.7)	77(10.2)			7(7.3)	75(9.8)			3(2.3)	79(10.8)			
Junior high school	30(28.0)	275(36.4)			30(31.3)	275(35.9)			24(18.5)	281(38.3)			
Senior high school	28(26.2)	170(22.5)			24(25.0)	174(22.7)			28(21.5)	170(23.2)			
University or college and above	44(41.1)	234(31.0)			35(36.5)	243(31.7)			75(57.7)	203(27.7)			
***Enabling variables***													
**Income monthly**			0.616	0.893			1.245	0.742				4.951	0.175
< 3000 RMB	23(21.5)	168(22.2)			20(20.8)	171(22.3)			26(20.0)	165(22.5)			
3000–4999	41(38.3)	280(37.0)			39(40.6)	282(36.8)			45(34.6)	276(37.7)			
5000–9999	33(30.8)	251(33.2)			28(29.2)	256(33.4)			50(38.5)	234(31.9)			
≧10,000	10(9.3)	57(7.5)			9(9.4)	58(7.6)			9(6.9)	58(7.9)			
**Time residing in Beijing**			4.047	0.256			0.982	0.806				6.555	0.088
< 1 year	14(13.1)	68(9.0)			11(11.5)	71(9.3)			24(18.5)	58(7.9)			
1-	19(17.8)	161(21.3)			17(17.7)	163(21.3)			30(23.1)	150(20.5)			
5-	30(28.0)	258(34.1)			33(34.4)	255(33.2)			42(32.3)	246(33.6)			
≧10	44(41.1)	269(35.6)			35(36.5)	278(36.2)			34(26.2)	279(38.1)			
**Plan to reside for a long time in Beijing**			4.316	0.038			1.747	0.186				49.602	< 0.001
No	30(28.0)	293(38.8)			30(31.3)	300(39.1)			37(28.5)	286(39.0)			
Yes	77(72.0)	463(61.2)			66(68.8)	467(60.9)			93(71.5)	447(61.0)			
**Have at least one child in Beijing**			4.726	0.030			0.071	0.789				28.977	< 0.001
No	70(65.4)	569(75.3)			70(72.9)	569(74.2)			106(81.5)	533(72.7)			
Yes	37(34.6)	187(24.7)			26(27.1)	198(25.8)			24(18.5)	200(27.3)			
**Employment status**			44.567	< 0.001			0.033	0.856				29.977	< 0.001
Formal work	49(45.8)	300(39.7)			38(39.6)	311(40.5)			87(66.9)	262 (30.6)			
Informal work	58(54.2)	456(60.3)			58(60.4)	456(59.5)			43(33.1)	471 (69.4)			
**Housing source**			0.152	0.004			6.106	0.013				0.806	0.421
Own house	22(20.6)	82(10.8)			19(19.8)	85(11.1)			17(13.1)	87(8.8)			
Rent	85(79.4)	674(89.2)			77(80.2)	682(88.9)			113(86.9)	646(91.2)			
**Insurance**			1.741	0.303			1.279	0.419				0.407	0.614
Uninsured	2(1.9)	35(4.6)			2(2.1)	35(4.6)			5(3.8)	32(4.8)			
Insured	105(98.1)	721(95.4)			94(97.9)	732(95.4)			125(96.2)	701(95.2)			
**Average daily working time**			22. 026	< 0.001			18.335	< 0.001				43.817	< 0.001
< 8 h	7(6.5)	24(3.2)			7(7.3)	24(3.1)			7(5.4)	24(3.8)			
8	69(64.5)	333(44.0)			60(62.5)	342(44.6)			90(69.2)	312(34.6)			
> 8	31(29.0)	399(52.8)			29(30.2)	401(52.3)			33(25.4)	397(61.7)			
***Health behavior***													
**Health promotion behaviors**													
**Do exercises**			23.561	< 0.001			19.064	< 0.001				29.872	< 0.001
No	38(35.5)	456(60.3)			35(36.5)	459(59.8)			55(42.3)	439(67.2)			
Yes	69(64.5)	300(39.7)			61(63.5)	308(40.2)			75(57.7)	294(32.8)			
**Acquire health knowledge**			15.584	< 0.001			1.362	0.279				24.476	< 0.001
No	32(29.9)	385(50.9)			41(42.7)	376(49.0)			48(36.9)	369(57.4)			
Yes	75(70.1)	371(49.1)			55(57.3)	391(51.0)			82(63.1)	364(42.6)			
**Health hazard behaviors**													
**Smoking**			1.153	0.283			0.328	0.567				13.121	< 0.001
No	92(86.0)	618(81.7)			81(84.4)	629(82.0)			104(80.0)	606(77.2)			
Yes	15(14.0)	138(18.3)			15(15.6)	138(18.0)			26(20.0)	127(22.9)			
**Drinking**			2.804	0.094			2.213	0.137				1.487	0.234
No	90(84.1)	677(89.6)			81(84.4)	686(89.4)			110(84.6)	657(87.5)			
Yes	17(15.9)	79(10.4)			15(15.6)	81(10.6)			20(15.4)	76(12.5)			
***Need variables***													
**Having chronic disease**			0.739	0.390			1.740	0.222				0.067	0.795
No	89(83.2)	602(79.6)			72(75.0)	619(80.7)			103(79.2)	588(80.2)			
Yes	18(16.8)	154(20.4)			24(25.0)	148(19.3)			27(20.8)	145(19.8)			
**Self-evaluation general health status**			1.290	0.525			1.391	0.499				2.435	0.296
Good	32(29.9)	193(25.5)			28(29.2)	197(25.7)			37(28.5)	188(25.6)			
Moderate	47(43.9)	332(43.9)			44(45.8)	335(43.7)			49(37.7)	330(45.0)			
Poor	28(26.2)	231(30.6)			24(25.0)	235(30.6)			44(33.8)	215(29.3)

**Table 4:** The main types of health education received and hope to receive by migrants in different gender

Types	Received n (%)		P*	Hope to receive n (%)		P*
	Male	Female		Male	Female	
**Child healthcare**	57(14.9)	79 (16.5)	0.528	172(44.9)	232(48.3)	0.316
**Occupation disease prevention and therapy**	73(19.1)	57(11.9)	0.003	141(36.8)	138(28.7)	0.012
**Antenatal, prenatal and postpartum healthcare**	39(10.2)	90 (18.8)	< 0.001	121(31.6)	186(38.8)	0.029
**CD prevention and therapy**	38(9.9)	69(14.4)	0.097	179(46.7)	243(50.6)	0.256
**NCD prevention and therapy**	35(9.1)	61(12.7)	0.049	223(58.2)	305(63.5)	0.111
**Adolescent healthcare**	33(8.6)	45(9.4)	0.699	135(35.2)	193(40.2)	0.136
**Aged healthcare**	24(6.3)	47(9.8)	0.061	161(42.0)	248(51.7)	0.005
**Menopause healthcare**	18(4.7)	38(7.9)	0.107	119(31.1)	193(40.2)	0.006
**Total**	207(54.0)	257(53.5)	0.719	230(60.1)	302(62.9)	0.390

***Note:*** There are overlaps in the number of migrants who received the different types of health education

* Chi-square independence test was used for each variable

Table 5 shows that the main types of health education received by migrants in different age groups. “Occupational disease prevention and therapy” (30.9%), “CD prevention and therapy” (15.5%), and “adolescent healthcare” (18.2%) were received by migrants in the age group of 18 to 24 years old more than other groups. “Antenatal, prenatal and postpartum healthcare” (24.4%) and “child healthcare” (22.8%) were received by migrants in the age group of 25 to 34 years old more than other groups. “NCD prevention and therapy” (15.5%), “aged healthcare” (15.5%) and “menopause healthcare” (10.3%) were received by migrants in the age group of 55 years old and above more than other groups.

**Table 5:** The main types of health education received by migrants in different ages

Types	Received n (%)					P*
	18–24	25–34	35–44	45–54	≧55	
**Child healthcare**	21(19.1)	72 (22.8)	30(14.2)	10(6.0)	3(5.2)	< 0.001
**Occupation disease prevention and therapy**	34(30.9)	53(16.8)	27(12.8)	13(7.7)	3(5.2)	< 0.001
**Antenatal, prenatal and postpartum healthcare**	12(10.9)	77 (24.4)	28(13.3)	6(3.6)	6(10.3)	< 0.001
**CD prevention and therapy**	17(15.5)	41(13.0)	30(14.2)	12(7.1)	7(12.1)	0.202
**NCD prevention and therapy**	7(6.4)	35(11.1)	26(12.3)	19(11.3)	9(15.5)	0.411
**Adolescent healthcare**	20(18.2)	29(9.2)	17(8.1)	8(4.8)	4(6.9)	0.004
**Aged healthcare**	5(4.5)	22(7.0)	17(8.1)	15(8.9)	9(15.5)	0.005
**Menopause healthcare**	4(3.6)	17(5.4)	16(7.6)	13(7.7)	6(10.3)	0.192
**Total**	84(76.4)	207(65.5)	97(46.0)	56(33.3)	20(34.5)	< 0.001

***Note:*** There are overlaps in the number of migrants who received the different types of health education

* Chi-square independence test was used for each variable

Table 6 shows that the types of health education desired to receive by migrants in different age groups. “Occupational disease prevention and therapy” (54.5%), “adolescent healthcare” (54.5%), and “antenatal, prenatal and postpartum healthcare” (50.9%) were desired to receive by migrants in the age group of 18 to 24 years old more than other groups. “NCD prevention and therapy” (37.6%), “aged healthcare” (81.0%) were wanted to receive by migrants in the age group of 55 years old and above more than other groups. “Child healthcare” (59.9%), “CD prevention and therapy” (53.8%), and “menopause healthcare” (45.2%) were wanted to receive by migrants respectively in the age groups of 25 to 34 years old, 35 to 44 years old, and 45 to 54 years old more than other groups.

**Table 6:** The main types of health education want to receive by migrants in different age

Types	Want to receive N (%)					P*
	18–24	25–34	35–44	45–54	≧55	
**Child healthcare**	55(50.0)	190 (59.9)	103(49.0)	40(23.8)	16(27.6)	< 0.001
**Occupation disease prevention and therapy**	60(54.5)	111(35.0)	53(25.2)	46(27.4)	9(15.5)	< 0.001
**Antenatal, prenatal and postpartum healthcare**	56(50.9)	154 (48.6)	65(31.0)	25(14.9)	7(12.1)	< 0.001
**CD prevention and therapy**	50(45.5)	151(47.6)	113(53.8)	77(45.8)	31(53.4)	0.425
**NCD prevention and therapy**	64(58.2)	170(53.6)	131(62.4)	118(70.2)	45(77.6)	< 0.001
**Adolescent healthcare**	60(54.5)	147(46.4)	73(34.8)	36(21.4)	12(20.7)	< 0.001
**Aged healthcare**	50(45.5)	128(40.4)	87(41.4)	97(57.7)	47(81.0)	< 0.001
**Menopause healthcare**	39(35.5)	98(30.9)	76(36.2)	76(45.2)	23(39.7)	0.039
**Total**	74(67.3)	204(64.4)	123(58.6)	97(57.7)	34(58.6)	0.330

***Note:*** There are overlaps in the number of migrants who received the different types of health education

* Chi-square independence test was used for each variable

Additionally, the top three pathways through which migrants want to acquire health information were television broadcasting (62.8%), internet (webpage and WeChat) (58.2%), and professionals and health managers (37.4%).

### Multivariate logistic regression model

The findings indicate that all of the full models were able to distinguish between migrants with receiving health education and those without receiving health education, and all predictors were statistically significant at the *P* < 0.001 level (Model I, χ2 = 186.467, *P* < 0.001; Model II, χ2 = 49.367, *P* < 0.001; Model III, χ2 = 39.895, *P* < 0.001; Model IV, χ2 = 90.941, *P* < 0.001). In the model summary, Model I explained between 19.6% (Cox and Snell R square) and 26.3% (Nagelkerke R square) of the variance in health education utilization as a whole. Model II explained between 5.6% (Cox and Snell R square) and 10.7% (Nagelkerke R square) of the variance in CD health education utilization as a whole. Model III explained between 4.6% (Cox and Snell R square) and 9.1% (Nagelkerke R square) of the variance in NCD health education utilization of migrants as a whole. Model IV explained between 10.1% (Cox and Snell R square) and 17.7% (Nagelkerke R square) of the variance in occupational disease health education utilization as a whole. (See Table 7).

**Table 7:** Model summary of health education utilization of migrants

	-2Log likelihood	Cox and Snell R Square	Nagelkerke R Square
**Model I**^**a**^	993.868	0.196	0.262
**Model II**^**b**^	587.575	0.056	0.107
**Model III**^**c**^	556.608	0.046	0.091
**Model IV**^**d**^	631.023	0.101	0.177

***Note:*** Model **I**: Binary logistic regression analysis of predictors of health education utilization of migrants in the past month

Model **II**: Binary logistic regression analysis of predictors of CD health education utilization of migrants in the past month

Model **III**: Binary logistic regression analysis of predictors of NCD health education utilization of migrants in the past month

Model **IV**: Binary logistic regression analysis of predictors of occupational disease health education utilization of migrants in the past month

^a^: χ2 = 186.467, *P* < 0.001

^b^: χ2 = 49.367, *P* < 0.001

^c^: χ2 = 39.895, *P* < 0.001

^d^: χ2 = 90.941, *P* < 0.001

Table 8 predicts the determinant factors of health education utilization for migrants (Model I) by multivariate logistic regression. Model I shows that the migrants with 25–34, 35–44, 45–54, above 55 years old in the past year were at 0.559-times (OR = 0.559, 95%-CI: 0.327, 0.957), 0.302-times (OR = 0.302, 95%-CI: 0.168, 0.542), 0.278-times (OR = 0.278, 95%-CI: 0.149, 0.519) and 0.232-times (OR = 0.232, 95%-CI: 0.107, 0.505) lower chances of receiving health education respectively, compared with 18–24 years old group. Migrants with education of university or college and above, senior high school, and junior high school were at 4.423-times (OR = 4.423, 95%-CI: 2.229, 8.774), 3.545-times (OR = 3.545, 95%-CI: 1.814, 6.929), and 2.129-times (OR = 2.129, 95%-CI: 1.414, 3.973) higher chances of receiving health education respectively in the past year, compared with migrants with education of primary school or below. Migrants who have at least one child in Beijing (OR = 1.901, 95%-CI: 1.290, 2.800), do exercises (OR = 1.989, 95%-CI: 1.454, 2719), have chronic disease (OR = 1.565, 95%-CI: 1.035, 2.366) were more likely to receive health education. Plan to reside for a long time in Beijing (OR = 0.674, 95%-CI: 0.479, 0.946), smoking (OR = 0.605, 95%-CI: 0.406, 0.902), were less likely to receive health education. Additionally, the chances of receiving health education decreased 43.4% in poor health status (OR = 0.566, 95%-CI: 0.365, 0.877) and 41.9% in moderate health status (OR = 0.581, 95%-CI: 0.391, 0.862), compared with self-evaluated good health status.

**Table 8:** Multivariate logistic regression analysis of predictors of health education utilization of migrants

Variables in the equation	Model I			
	B (SE)	Wald	OR[95%-CI]	***P***
***Predisposing variables***				
Age (Ref = 18–24)				
25–34	− 0.581(0.274)	4.503	0.559[0.327, 0.957]	0.034
35–44	− 1.198(0.298)	16.131	0.302[0.168, 0.542]	< 0.001
45–54	−1.279(0.318)	16.211	0.278[0.149, 0.519]	< 0.001
≧55	−1.461(0.397)	13.535	0.232[0.107, 0.505]	< 0.001
Education level (Ref = Primary school or below)				
Junior high school	0.756(0.318)	5.642	2.129[1.141,3.973]	0.018
Senior high school	1.266(0.342)	13.703	3.545[1.814,6.929]	< 0.001
University or college and above	1.487(0.349)	18.097	4.423[2.229,8.774]	< 0.001
***Enabling variables***				
Plan to reside for a long time in Beijing (Ref = No)				
Yes	−0.395(0.173)	5.184	0.674[0.479, 0.946]	0.023
Have at least one child in Beijing (Ref = No)				
Yes	0.642(0.198)	10.561	1.901[1.290,2.800]	0.001
***Health behavior variables***				
Do exercises (Ref = No)				
Yes	0.687(0.160)	18.546	1.989[1.454,2.719]	< 0.001
Smoking (Ref = No)				
Yes	−0.502(0.204)	6.075	0.605[0.406,0.902]	0.014
***Need variables***				
Having chronic disease (Ref = No)				
Yes	0.448(0.211)	4.500	1.565[1.035,2.366]	0.034
Self-evaluation general health status **(**Ref = good**)**				
General	−0.543(0.202)	7.261	0.581[0.391,0.862]	0.007
Poor	−0.569(0.223)	6.476	0.566[0.365,0.877]	0.011
Constant	0.096 (0.412)	0.054	1.101	0.816

Abbreviation: *B* Unstandardized regression coefficient; *SE* standard error; *OR* odds ratio; *CI* confidence interval; *Ref* reference category

Model I: Multivariate logistic regression analysis of predictors of health education receipt by migrants

Table 9 predicts the determinants of CD, NCD, and occupational disease health education utilization for migrants (Model II, Model III, and Model IV) by multivariate logistic regression. Model II shows that the chances of receiving CD health education decreased 71.0% in average daily working time more than 8 h (OR = 0.290, 95%-CI: 0.113, 0.744), compared with migrants with average daily working time less than 8 h. Additionally, migrants who do exercises (OR = 2.204, 95%-CI: 1.423, 3.415), acquire health knowledge (OR = 1.954, 95%-CI: 1.236, 3.091) were more likely to receive CD health education. Model III indicates that divorced or widowed migrants were at 4.448-times (OR = 4.448, 95%-CI: 1.193, 16.584) higher chance of receiving NCD health education, compared with unmarried migrants. The chances of receiving NCD health education decreased 74.6% in average daily working time more than 8 h (OR = 0.254, 95%-CI: 0.098, 0.655), compared with migrants with average daily working time less than 8 h. Additionally, migrants who do exercises (OR = 2.436, 95%-CI: 1.555, 3.861), were more likely to receive NCD health education. Model IV indicates that the chances of receiving occupational disease health education decreased 58.3% in married (OR = 0.417, 95%-CI: 0.264, 0.657), and 68.5% in average daily working time more than 8 h (OR = 0.315, 95%-CI: 0.120, 0.827), compared with unmarried migrants and average daily working time less than 8 h respectively. Migrants who have formal work (OR = 2.001, 95%-CI: 1.245, 3.217), do exercises (OR = 1.827, 95%-CI: 1.222, 2.734), were more likely to receive occupational disease health education.

**Table 9:** Multivariate logistic regression analysis of predictors of three types health education utilization of migrants

Variables in the equation	Model II				Model III				Model IV			
	B (SE)	Wald	OR[95%-CI]	***P***	B (SE)	Wald	OR[95%-CI]	***P***	B (SE)	Wald	OR[95%-CI]	***P***
***Predisposing variables***												
Marital status (Ref = Unmarried)												
Married					0.501(0.302)	2.756	1.650 [0.913, 2.981]	0.097	−0.876(0.232)	14.216	0.417 [0.264, 0.657]	< 0.001
Divorced/Widowed					1.492(0.671)	4.941	4.448 [1.193, 16.584]	0.026	−0.519(0.806)	0.415	0.595 [0.123, 2.888]	0.520
Employment status (Ref = Informal work)												
Formal work									0.694(0.242)	8.195	2.001 [1.245, 3.217]	0.004
***Enabling variables***												
Average daily working time (Ref = < 8 h)												
8	−0.356(0.461)	0.595	0.701 [0.284, 1.729]	0.440	−0.422(0.465)	0.825	0.656 [0.264, 1.630]	0.364	−0.426(0.481)	0.783	0.653 [0.255, 1.677]	0.376
> 8	−1.237(0.480)	6.634	0.290 [0.113, 0.744]	0.010	−1.372(0.484)	8.037	0.254 [0.098, 0.655]	0.005	−1.156(0.493)	5.495	0.315 [0.120, 0.827]	0.019
***Health behavior variables***												
Do exercises (Ref = No)												
Yes	0.790(0.223)	12.519	2.204 [1.423,3.415]	< 0.001	0.890(0.229)	15.111	2.436 [1.555, 3.861]	< 0.001	0.603(0.205)	8.610	1.827 [1.222,2.734]	0.003
Acquire health knowledge (Ref = No)												
Yes	0.670(0.234)	8.203	1.954 [1.236,3.091]	0.004								
Constant	−2.087 (0.480)	18.905	0.124	< 0.001	−2.188 (0.535)	16.738	0.112	< 0.001	−0.799 (0.527)	2.297	0.130	0.450

***Note:*** Model **II**: Multivariate logistic regression analysis of predictors of CD health education utilization of migrants in the past month

Model **III**: Multivariate logistic regression analysis of predictors of NCD health education utilization of migrants in the past month

Model **IV**: Multivariate logistic regression analysis of predictors of occupational disease health education utilization of migrants in the past month

## Discussion

This study attempted to describe the differences between the needs and utilization of health education, and assess the major determinants associated with the health education utilization for migrants in urban-rural fringe areas of Beijing, to better facilitate their health education utilization.

### Utilization and needs of health education

Previous researches indicated that the advantage of “healthy migrant effect” (first-generation migrants are often healthier with lower overall morbidity and mortality than local-born populations) will diminish dramatically, particularly in middle age [29, 30], together with demanding work schedules, poor working and living environment, insufficient health literacy, and negative attitudes toward the health preventive behaviors. Our research revealed that though many migrants were aware of significance of health education, and expressed a desire to gain access to health information for enhancing their well-being, yet low utilization rate lingered and only 34.5% migrants received health education in the past year. It was self-evident that most of the migrants investigated were middle-aged, and they had age-appropriate health education needs, such as antenatal, prenatal and postpartum healthcare, aged healthcare, and NCD prevention and therapy. Furthermore, with the evolution of migration model, migrants should take the responsibilities for caring for their child(ren) and parent(s), thus they have relatively high needs of child, adolescent, and aged healthcare [14]. It also seems strange that there were low rates of occupational disease health education utilization and needs actually, which are consistent with the previous study in Xi’an [20]. Furthermore, there might be a reason to explain the low needs of occupational disease health education among migrants. Different from acute occupational diseases (occupational allergic contact dermatitis, occupational poisoning) that always occur after a relatively brief exposure, the common chronic occupational diseases (pneumoconiosis, musculoskeletal disorders, psychological stress at work, occupational tumors) which occupy the majority of occupational diseases, only occur after prolonged exposure to relevant hazards [31, 32]. Migrants with low health literacy, unstable job, and limited knowledge of occupational hazards, would not pay enough attention to occupational diseases, even if chronic occupational diseases have occurred.

### Determination factors of whole health education utilization

The rate of health education utilization was higher in the groups of 18 to 24 and 25 to 34 years old than that in other three age groups, particularly in the first age group. The result was similar to a previous China-based study that migrants in the group of 25 to 34 years old have higher rate of health education utilization than other age groups, but the rate of migrants in age group of 18 to 24 years old was opposite [33]. There might be two factors for the differences. Firstly, the second-generation migrants with higher education level and relatively stable working condition, were born after 1980, had better health literacy and could acquire reliable health information from various sources, compared with the first-generation migrants [34]. Meanwhile, this research also indicated that migrants with high education level and regular exercises had a higher likelihood of receiving health education. The prevalence of health literacy was related to health knowledge, health decisions, health behaviors and health outcomes of the population [35–37]. On the contrary, low education level, accompanied by low literacy and health awareness, pose difficulties and barriers in understanding complex health-related information, health practices and outcomes [36]. Secondly, selection bias would influence the results due to insufficient sample size of migrants in age group of 18 to 24 years old. In the future, migrants in this age group should be studied in terms of their health education utilization behaviors and influencing factors as a unique group.

### Determination factors of three types of health education utilization

Gaps exist between the needs and utilization of three types of health education for general migrants population investigated, including CD, NCD and occupational disease. Our study focuses on the determinations factors of three types of health education utilization.

For migrants, we observed that both average daily working time of enabling variables and do exercises of health behavior variables contributed significantly to the variances in three types of health education utilization. Migrants who worked more than 8 h daily and not to do exercises were less likely to use three types of health education than migrants with working time less than 8 h and do exercises. Put another way, the heavy workloads and poor health awareness for migrants reduce the opportunity to receive health information. Normally, people who do not exercise regularly are lacking of health awareness to access health education. Additionally, consistent with the previous research [33], migrants with formal work are more likely to receive occupational disease health education than those with informal work. In accordance with state regulations, employers have the responsibility to provide regular training, to educate their employees about occupational hazards, and to require them to strictly abide by safety rules. While, migrants are overwhelmingly employed in 3Ds jobs (dirty, dangerous and degrading) in worldwide, covering the service sector, production, construction and maintenance, transportation, which have more health-related risks and access less health education compared with other industries [22, 38–40]. Meanwhile, the informal and temporary working status, long working time and situational stress cut down the needs of acquiring health information, especially on occupational disease for migrants.

### Device of new health education tools

Advances of information technology witness that smartphones and internet have become an integral part of our lives, and are widely used in health information research. The report of *“Internet adoption, social media usage, and smartphone ownership rates in 37 countries across the world in 2017”* from Pew research center revealed that the rates of internet use, smartphone ownership and social media use were 71, 68 and 60% respectively in China [41]. Different from traditional text-based health education tools, including brochures, leaflets, newspapers, web-based social media tools offer more convenient and effective methods of delivering health information [42]. Take the WeChat application as an example, as a free instant messaging application for smartphones, it plays an important part in modern lifestyles. WeChat can serve as a bridge between information technology and frequent multimedia messages to provide health support and management through the communication and transmission of voices, texts, pictures and videos over great distances [43]. Information related to the prevention and treatment of various diseases can be acquired and requested at any time via such applications [44, 45]. The increasing number of smartphones in China provides a mobile platform for delivering health education. As in our study, 58.2% migrants wanted to acquire health information via the internet. Therefore, mobile health interventions strategies have enormous potentials as an educational tool for behavioral change to further control the spread of epidemics, and development of the chronic disease for migrants.

### Limitations

The study has several limitations. Firstly, cross-sectional survey cannot be determined the time-effect and causality accurately compared with the cohort study, and cannot evaluate the effects of health education compared with the intervention study. Secondly, recall biases on self-report might underestimate the information on health education utilization. Thirdly, health education utilization behaviors were measured as a dichotomous variable (the received or did not receive of health education), rather than measured by the intensity of received health education. Finally, although the questionnaire was designed according to the previous theories and experiences, several significant potential determination factors may not be considered in the model, such as community resource factors. Future research is needed to explain the dynamic and cyclical causal relationships of Anderson’s health service utilization behavioral model by identifying more variables.

## Conclusion

The findings of the survey contribute to our understanding of the health education utilization, determination factors, and needs of health education among Chinese migrants. There were certain gaps between the needs and utilization in different types of health education. Compared with the first-generation migrants, second-generation migrants had higher rate of health education utilization. Additionally, average daily working time of enabling variables and do exercises of health behavior variables in the Anderson health service utilization behavioral model, was a dominant predictor of three types diseases of health education utilization, including CDs, NCDs and occupational diseases. Many migrants desired for more health education information from internet. In the next step, we should focus on the health education utilization for migrants with heavy workload and low education level.

The findings of this research might be useful for establishing basic public health service network. It also suggests that policy makers should take feasible policies and measures to overcome obstacles and to break down barriers for migrants, including fully implementing health education intervention strategies and policies, providing multiple health information channels, ensuring easy and equitable access to health education. In the future, more comprehensive studies should be carried out to evaluate the efficiency of health intervention strategies to improve the acquisition and utilization of basic public health services for migrants.

## Supplementary Information

**Additional file 1.**

## Data Availability

The datasets generated and/or analyzed during the current study are available from the corresponding author on reasonable request.

## Abbreviations

SARS: Severe Acute Respiratory Syndrome
CD: Communicable disease
NCD: Chronic non-communicable disease
RMB: *Renminbi*
CHSIs: Community health service institutions
PEN: Predisposing, enabling, and need variances
