# Supplementary material for: Health education services utilization and its determinants among migrants: a cross-sectional study in urban-rural fringe areas of Beijing, China
Source: BMC Fam Pract. 2021 Jan 16;22:23. doi: 10.1186/s12875-021-01368-1 (PMC7811350; doi:10.1186/s12875-021-01368-1)
Supplement: Supplementary file 1 — Additional file 1. [file 12875_2021_1368_MOESM1_ESM.doc]

**Additional File 1.**

**An questionnaire investigation of health education services utilization and needs for migrants in** **urban-rural fringe areas in Beijing**

Number ______________

District _______________

Street _______________

Community _______________

Investigate date: _____________ Signature of investigator:______________

Check date: _____________ Signature of scrutator: _______________

**Dear Migrants:**

The research is a part of Beijing Philosophy and Social Science Planning Project, a population-based cross-sectional survey on risk factors of health status for migrants and sponsored by Capital Medical University. The questionnaire is only used to investigate your utilization of health education and needs. Your thoughts are important to improve the Basic Public Health Service Program. Please take a few minutes to fill in the following questions carefully. We assure you that your answers will be kept strictly confidential.

Thank you for your support.

Health education utilization research team

June 2016

Note:

1. All respondents were at least 18 years old.

2. Inclusion criteria: inter-provincial migrants residing or working in the sampling regions (for no less than six months).

3. Exclusion criteria: migrants who were not able to respond, those with mental health issues, and tourists in Beijing.

(Please mark the “√” under the numbers, e.g. ①, ②, or write the content on the “_____” following the request of each question)

**Part 1. Social demography characteristic**

1. Sex: ① Male ② Female

2. Birth date: Year ________ Month________

3. Educate level:

①University or college and above

②Senior high school

③Junior high school

④Primary school or below

4. Marital status:

①Unmarried

②Married

③Divorced/widowed

5. Ethnicity:

①Han ethnic

②Minorities

6. What is the type of your “*Hukou*” system?

①Non-agricultural

②Agricultural

7. How long have you live in Beijing? ________ Year

8. Do you plan to reside for a long time in Beijing?

①Yes

②No

9. What is your employment status in Beijing?

①Formal work

②Informal work

10. How much money did you earn monthly in the past year?

①<3000 RMB

②3000-4999

③5000-9999

④≧10000

11.Do you have any form of health insurance?

①Yes

②No

12. How long did you work average day in the past year? _____Hours

13. Did you have at least one child living with in Beijing in the past year?

①Yes

②No

14. What is the type of house you live?

①Own house

②Rent

**Part 2. Health behavior and health status**

15. Did you often do exercises daily time in the past year? Note: at least 30 minutes daily, 5 days weekly.

①Yes

②No

16. Did you often acquire health knowledge in the past year?

①Yes

②No

17. Did you often smoke in the past year?

①Yes

②No

18. Did you often drink in the past year?

①Yes

②No

19. Do you have any form of chronic disease?

①Yes

②No

20. How did you evaluate your general health status in the past year?

①Good

②General

③Poor

**Part 3. Health education utilization**

21. Have you received any form of health education in the past year?

Note: Including receipt of "materials of health education", "health education bulletin board", "public health consultation", "health knowledge lecture", and "individualized health education" by population from CHSCs.

①Yes

②No

22. What was/were the type/types of health education have you received in the past year?

①Occupational disease prevention and therapy

②Child healthcare

③Antenatal, prenatal and postpartum healthcare

④Communicable disease prevention and therapy

⑤Non communicable disease prevention and therapy

⑥Adolescent healthcare

⑦Menopause healthcare

⑧Aged healthcare

⑨Other___________

**Part 4. Health education utilization needs**

23. What is/are the type/types of health education that you want to receive in the future?

①Occupational disease prevention and therapy

②Child healthcare

③Antenatal, prenatal and postpartum healthcare

④Communicable disease prevention and therapy

⑤Non communicable disease prevention and therapy

⑥Adolescent healthcare

⑦Menopause healthcare

⑧Aged healthcare

⑨Other___________

Thank you very much for taking the time to complete this survey. Your feedback is valued and very much appreciated.
